# The complete mitochondrial genome of a deep sea ophiuroid of the genus *Amphiura* (Ophiuroidea: Amphiuridae)

**DOI:** 10.1080/23802359.2019.1679047

**Published:** 2019-10-23

**Authors:** Jieying Na, Dongsheng Zhang, Hong Cheng, Juan Yang, Ruiyan Zhang, Wanying Chen, Chunsheng Wang

**Affiliations:** a Key Laboratory of Marine Ecosystem and Biogeochemistry, State Oceanic Administration & Second Institute of Oceanography, Ministry of Natural Resources, Hangzhou, PR China;; b School of Marine Sciences, China University of Geosciences(Beijing), Beijing, PR China;; c School of Oceanography, Shanghai Jiao Tong University, Shanghai, PR China;; d State Key Laboratory of Satellite Ocean Environment Dynamics, Hangzhou, PR China

**Keywords:** *Amphiura*, mitogenome, deep sea, seamount, west Pacific

## Abstract

*Amphiura* is a widely distributed genus of Ophiuroidea from various environment, play an important role in evolution. Here, we reported a complete mitochondrial genome of *Amphiura* sp. which was collected from a deep sea seamount in the West Pacific. The mitogenome is 15,457 in length, including 13 protein-coding genes, 2 rRNA genes, and 22 tRNA genes. All genes are arranged in the same order of published mitogenomes in the same genus. The phylogenetic analysis support monophyly of the family Amphiuridae but not for the genus *Amphiura*.

*Amphiura* is one of the largest genera of Ophiuroidea reported from various environments (Stöhr and Segonzac 2005; Cecchetto et al. 2017; Alitto et al. 2018). Many species from this genus have been studied well about their biology, physiology and life history (Czarkwiani et al. 2016; Delroisse et al. 2017). However, very few studies have been published about *Amphiura* from deep sea (Hendler and Tran 2001). Recently, studies focussed on mitochondrial genome revealed that Ophiuroidea has undergone more complex gene rearrangements than other classes of echinoderms (Galaska et al. 2019; Lee et al. 2019). In this paper, the complete mitochondrial genome from a deep-sea *Amphiura* sp. was characterized in order to provide genetic information for further phylogenetic analysis.

The specimen was collected from the Weijia Guyot (156°28'E, 12°37'N, 1995 m depth) using ROV *HAIMA*, and was identified as *Amphiura* sp. based on the morphological characters. The specimen (RSIO41201) and its DNA (DNASIO41201) are deposited in the Repository of the Second Institute of Oceanography. Genomic DNA was extracted with a DNeasy Blood & Tissue Kit (QIAGEN, Valencia, CA), COI and 16S were first amplified and sequenced with specific primers (Palumbi et al. 1991; O'Hara et al. 2014), then the two partial mitochondrial genome sequences (COI-16S and 16S-COI) were amplified by long PCR specific primers designed according to COI and 16S sequences, then sequenced by NGS on Illumina HiSeq XTen platform (Illumina Inc. San Diego, CA). About 1G clean data of each partial sequences were de novo assembled using SPAdes-3.12 (Nurk et al. 2013). The remaining gaps were amplified with the species-specific primers designed according to the obtained sequences. A contig with a length of 15,457 bp was considered as the mitogenome (GenBank accession number: MN296491), on which protein-coding genes (PCGs), ribosomal RNA (rRNA) genes, and transfer RNA (tRNA) genes were annotated with MITOS2 WebServer (Donath et al. in press). The boundaries of PCGs were manually adjusted by comparison with published Ophiuroid mitogenomes. Alignments of the 13 PCGs were carried out by Geneious Prime with default settings. Phylogenetic relationships were estimated using maximum likelihood, which performed by raxmlGUI 1.5b2 (Silvestro and Michalak 2012) using a selection of ML + thorough bootstrap with 1000 replicates and the GTR + I + G model of substitution for the nucleotide dataset. FigTree v1.4.3 (Rambaut 2016) was used to visualize tree files.

The complete mitogenome of *Amphiura* sp. contains 13 PCGs, two rRNA genes, and 22 tRNA genes. All PCGs start with ATG, except for ND6, which use GTG as the start codon, and CYTB use ATT as the start codon; most PCGs end with TAA codon, with the exception of ND6, CYTB, ND2, and ND1, which terminate with TAG codon, and COX2 terminate with TA codon (Figure 1).

**Figure 1.**
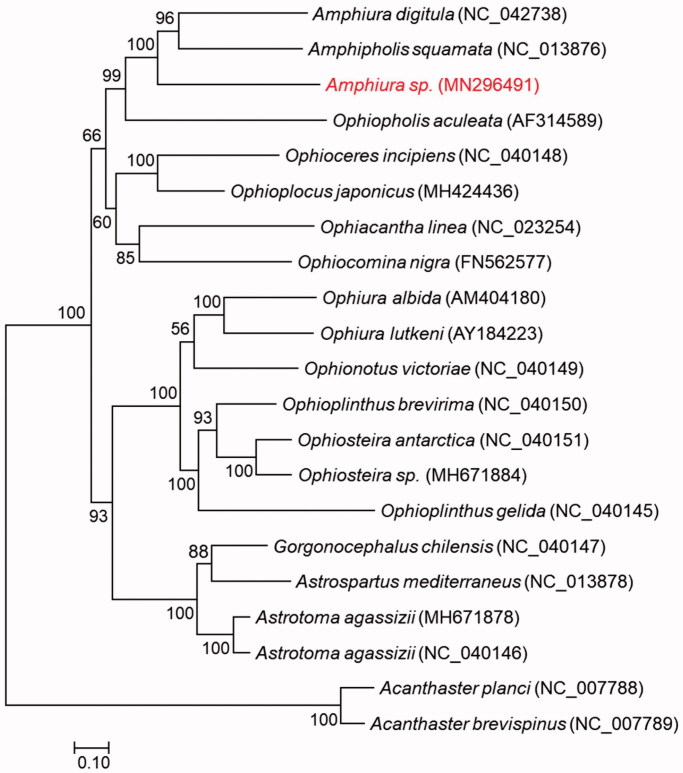
Phylogenetic tree of maximum-likelihood (ML) method based on the concatenated amino acid (AA) sequences of 13 PCGs of *Amphiura* sp. (MN296491) and 18 other ophiuroids.

The gene orders are identical to the two published mitogenomes from the same genus, further confirming the conservative gene order among congeners (Lee et al. 2019). Phylogeny inference with maximum likelihood robustly supports the monophyletic Amphiuridae but not to the genus *Amphiura* with *Amphipholis squamata* located between our species and *Amphiura digitula*.
